# ALCAM (CD166) Expression and Serum Levels in Pancreatic Cancer

**DOI:** 10.1371/journal.pone.0039018

**Published:** 2012-06-20

**Authors:** Michael Tachezy, Hilke Zander, Andreas H. Marx, Phillip R. Stahl, Florian Gebauer, Jakob R. Izbicki, Maximilian Bockhorn

**Affiliations:** 1 Department of General, Visceral and Thoracic Surgery, University Medical Center Hamburg-Eppendorf, Hamburg, Germany; 2 Institute of Pathology, University Medical Center Hamburg-Eppendorf, Hamburg, Germany; University of Munich, Germany

## Abstract

**Background:**

This study was conducted to evaluate the expression of the activated leukocyte cell adhesion molecule (ALCAM) in pancreatic cancer (PAC) and to determine whether or not the ectodomain shedding of ALCAM (s-ALCAM) could serve as a biomarker in the peripheral blood of PAC patients.

**Material and Methods:**

Tissue specimens and blood sera of patients with PAC (n = 264 and n = 116, respectively) and the sera of 115 patients with chronic pancreatitis (CP) were analyzed via ALCAM immunohistochemistry and s-ALCAM ELISA tests. Results were correlated with clinical, histopathological, and patient survival data (Chi-square test, Kaplan-Meier analysis, log-rank test, respectively).

**Results:**

ALCAM was expressed in the majority of PAC lesions. Immunohistochemistry and serum ELISA tests revealed no association between ALCAM expression in primary tumors or s-ALCAM and clinical or histopathological data. Neither ALCAM nor s-ALCAM showed a significant impact regarding overall survival (p = 0.261 and p = 0.660, respectively). S-ALCAM serum levels were significantly elevated compared to the sera of CP patients (p<0.001). The sensitivity of s-ALCAM in detecting PAC was 58.6% at a specificity of 73.9% (AUC = 0.69).

**Conclusions:**

ALCAM is expressed in the majority of PAC lesions, but statistical analysis revealed no association with clinical or pathological data. Although significantly elevated in patients with PAC, the sensitivity and specificity of the s-ALCAM serum quantification test was low. Therefore, its potential as a novel diagnostic marker for PAC remains elusive and further investigations are required.

## Introduction

Since most patients with pancreatic adenocarcinoma (PAC) present in advanced stages of the disease, the incidence of PAC is nearly equal to its mortality. Even in the curative setting, which only applies to a subset of patients, oncological long-term survival has not significantly improved over recent years (reported median survival of between 14 and 22 months); most of the tumors recur locally or at distant sites [1], [2]. Unfortunately, improvements in (neo-) adjuvant and even palliative treatment regarding recurrence and survival are still disappointing; none of the recently tested targeted therapies were very promising in clinical trials [3], [4], [5], [6].

As a consequence, two main goals must be achieved: first, new biochemical tests for the early detection, monitoring and prognosis of PAC should be developed. In addition, these could help to distinguish between malignant and benign pancreatic lesions, such as chronic pancreatitis (CP). In fact, there are still no established or recommended serum markers for the diagnosis or prognosis of PAC in routine use [7], [8]. Secondly, potential innovative targets for biological therapies must be identified to improve the survival of patients with PAC.

In recent times, the theory of the hierarchical organization of tumor cells was extensively investigated, supporting the cancer stem cell hypothesis [9], [10], [11], [12], [13]. These cells might be potential therapeutic biologic targets and prognostic markers. Several authors have identified putative stem cell markers for intestinal as well as PAC, namely CD133, CD44, and CD166, the activated leukocyte cell adhesion molecule (ALCAM) [10], [14], [15], [16], [17]. The latter is a highly conserved 110 kDa multidomain transmembrane type 1 glycoprotein of the immunoglobulin superfamily. This molecule mediates homotypic and heterotypic interactions between cells [18], [19]. It plays a role in the development of different tissues, for example in neurogenesis and haemotopoiesis, and it participates in the mechanisms of the immune response [20], [21], [22]. As with other membrane proteins, ALCAM represents a potential target for therapy and its utility as a drug target structure may be further enhanced by ligand-induced endocytosis [23]. Moreover, a recently described internalizing single-chain anti-ALCAM antibody has the potential to deliver therapeutic agents into cancer cells [23], [24].

Several studies reported its potential as a biomarker for different tumor entities, such as melanoma, pancreatic and ampullary adenocarcinoma, and colorectal, gynecological and neuroendocrine carcinomas. Its expression is associated with diverse outcomes in different tumors [17], [18], [19], [20], [25], [26], [27], [28], [29], [30], [31], [32]. Furthermore, the extracellular domain of ALCAM (s-ALCAM) is shed by metalloproteases (for example, ADAM 17), functions as an active messenger and interacts with surrounding tissues [33]. After cleavage from the tumor cell surface, s-ALCAM can be detected in the blood serum. An increased levels of s-ALCAM expression was observed in ovarian, breast and esophageal cancer patients compared to healthy controls [30], [33], [34], [35], [36]. In addition, studies with small patient samples showed an elevation of s-ALCAM expression in the sera of patients with PAC [37], [38].

We conducted the present study to investigate the association between ALCAM expression in a large number of primary PAC lesions and clinical and histopathological data and its potential prognostic value. Furthermore, we determined the preoperative s-ALCAM serum levels of patients with PAC and CP and evaluated its significance as a diagnostic and prognostic marker.

## Results

### Characteristics of the Patients

Pancreatic cancer tissue specimens from 264 patients aged 33 to 87 years (median 63 years) and blood sera from 116 PAC patients aged between 33 and 92 years (median 64 years) and 115 CP patients aged between 31 und 79 (median 51 years) were included in this study. All patients were surgically treated between 1993 and 2006 in the Department of General, Visceral and Thoracic Surgery of the University Medical Center Hamburg-Eppendorf, Germany.

The operation methods used were the classic Whipple procedure, pylorus-preserving pancreaticoduodenectomy, subtotal or total pancreatectomy in cases of PAC and organ-preserving resection methods in cases of CP.

The median follow-up time of the patients included in the survival analysis was 14 months (range 0–193 months) and the median overall survival (OS) was 15 months (95% CI 13.2–16.7 months). Twenty-nine (10.5%) patients died within the first 30 days after surgery.

### ALCAM Expression in PAC and its Correlation with Clinical and Histopathological Characteristics

**Figure 1:**
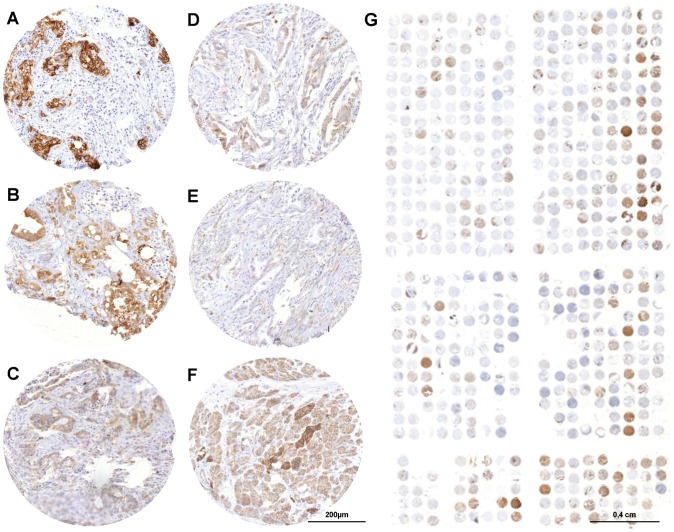
Representative immunohistochemical ALCAM stains of primary pancreatic cancer (PAC) lesions. (A) and (B) strong ALCAM expression, (C) and (D) medium and (E) no expression. (F) Healthy pancreatic tissue. (G) Complete scan of the PAC tissue microarray.

A total of 192 (78.6%) primary PAC tumor samples were interpretable in our tissue microarryay (TMA) analysis. Reasons for non-informative cases (52; 21.3%) included a complete lack of tissue samples or the absence of unequivocal cancer tissue in the TMA sections. Analysis of healthy pancreatic tissue revealed a weak or intermediate ALCAM expression (+/++) in normal acinar or ductal pancreatic cells (n = 10, Figure 1F). The staining pattern of the ALCAM immunohistochemistry shows a predominant membranous expression of the ALCAM molecule. Although some cytoplasmatic staining was occasionally seen, this was always associated with a much greater level of staining in the membranes. Thirty-eight per cent of the tumors showed a low level of ALCAM expression, 44% medium and 18% a high level of ALCAM expression (Figure 1A–1E, see Material and Methods for criteria). The tumors show a heterogeneous staining pattern inside the cancerous lesions (Figure 1A–1E).

The histopathological findings of the interpretable tumors are summarized in Table 1. The expression of ALCAM showed no association with clinical or histopathological parameters such as age, sex, tumor stage (UICC 6^th^ classification) or tumor grade (G).

**Table 1:** Association between the clinical and pathological parameters and the immunohistochemical ALCAM status of the primary tumor and ALCAM serum (s-ALCAM) levels.

			ALCAM IHC primary tumour																		Serum ALCAM level									
	Total		ALCAM low				ALCAM medium				ALCAM high							p-value		Total	Low (<42 ng/ml)				High (≥42 ng/ml)				p-value	
			(n = 72, 37%)				(n = 84, 44%)				(n = 36, 19%)										(n = 62, 75%)				(n = 20, 25%)					
Mean Age, years (range)			62.3			(33–83)	63.2		(33–87)		62.9				(43–82)			0.804			64.0		(31–92)		62.1		(48–76)		0.409	
Sex																														
Male	108		39			(36%)		58		(54%)			11		(10%)					46		36	(78%)		10		(22%)			
Female	84		33			(39%)		26		(31%)			25		(30%)			0.000		36		26	(72%)		10		(28%)	0.608		
Tumour Staging (UICC 6^th^ edition)																														
Ia°	6		2			(33%)		4		(67%)			0			(0%)				3	3		(100%)		0		(0%)			
Ib°	26		8			(31%)		8		(31%)			10			(38%)				9	5		(56%)		4		(44%)			
IIa°	41		17			(41%)		18		(44%)			6			(15%)				14	11		(79%)		3		(21%)			
IIb°	97		38			(39%)		44		(45%)			15			(16%)				43	31		(72%)		12		(28%)			
III°	10		3			(30%)		5		(50%)			2			(20%)				6	6		(100%)		0		(0%)			
IV°	12		4			(33%)		5		(42%)			3			(25%)		0.408		7	6		(86%)		1		(16%)	0.346		
Tumour grading																														
1 and 2		106		38	(36%)		46		(44%)			22		(21%)					58		46		(79%)	12		(21%)				
3		86		34	(40%)		38		(44%)			14		(16%)			0.710		24		16		(67%)	8		(33%)				0.264

**Figure 2:**
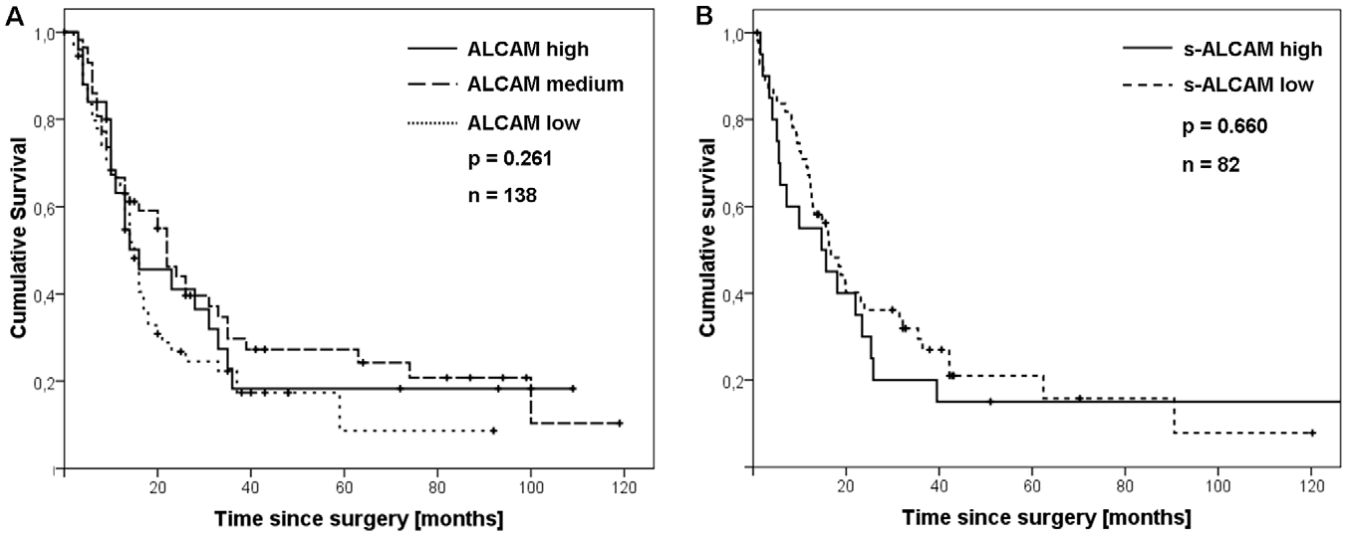
Kaplan-Meier overall survival analysis. (A) Immunohistochemical ALCAM staining of primary pancreatic cancer specimens and (B) low and high s-ALCAM serum levels (patients who died during the first 30 days after surgery were excluded).

The overall survival curves plotted by the Kaplan-Meier analysis did not reveal a significant difference between low, medium or high ALCAM-expression patients (p = 0.261, Figure 2A). Due to this, no multivariate analysis was performed.

### S-ALCAM in Blood Serum

**Figure 3:**
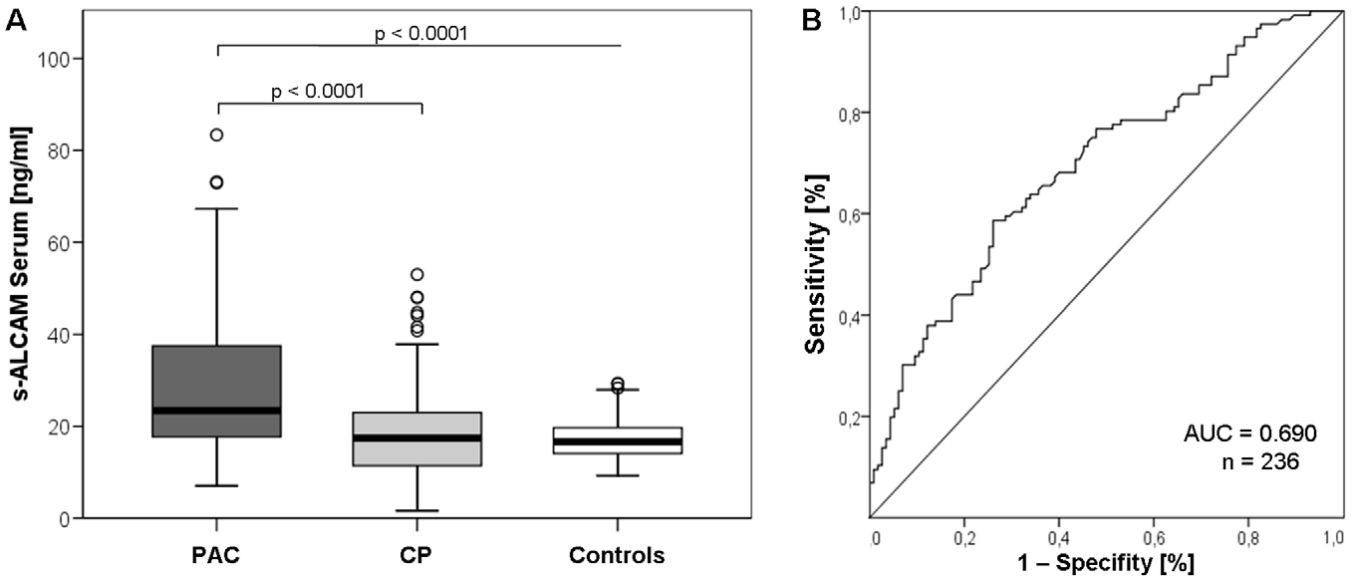
S-ALCAM serum analysis. (A) s-ALCAM serum levels of the patients with pancreatic cancer (PAC) and chronic pancreatitis (CP) and healthy control blood donors (p<0.001). (B) Receiver operating characteristic (ROC) curves of s-ALCAM for the diagnosis of PAC versus CP patients.

The s-ALCAM values were significantly elevated in the blood serum of PAC patients (n = 116, mean 29.4 ng/ml, standard deviation (SD) 1.1 ng/ml, p<0.001) compared to CP patients (n = 115, mean 18.2 ng/ml, SD 1.0 ng/ml) and healthy blood donors (n = 128, mean 21.1 ng/ml, SD 0.7 ng/ml, Figure 3A).

Receiver operating characteristic curves were used to establish the sensitivity-specificity relationship for s-ALCAM (Figure 3B). The cut-off level determined by the Youden index was 22 ng/ml. The AUC was 0.690. The sensitivity of s-ALCAM in detecting PAC was 58.6% at a specificity of 73.9% compared to patients with CP.

In order to determine the impact of elevated s-ALCAM levels on patients with PAC, continuous and categorical analyses were perfomed.

No significant differences were found regarding sex (female 33.6 ng/ml, male 31.3 ng/ml, p = 0.649), age (<64 years 33.0 ng/ml, >64 years 33.0 ng/ml; p = 0.775), UICC stage (Ia 24.1 ng/ml, Ib 36.0 ng/ml, IIa 26.8 ng/ml, IIb 36.4 ng/ml, III 19.0 ng/ml; IV 28.1 ng/ml; p = 0.277) and tumor cell grading (G1 and G2 31.1 ng/ml, G3 35.0 ng/ml; p = 0.479).

We defined different cut-off values for the categorical analysis of the s-ALCAM data. With none of them, (25^th^ percentile, median and 75^th^ percentile, the ‘optimal cut-off value’, determined using the Youden-index for discrimination of the UICC classification and tumor cell grading) a significant association with clinical or histopathological parameters was calculated.

To illustrate this, Table 1 presents the analysis with a cut-off value of the 75^th^ percentile (<42.3 ng/ml s-ALCAM, <75^th^ percentile; Table 1). The overall survival curves plotted by the Kaplan-Meier analysis showed no significant differences between the low and high s-ALCAM groups (p = 0.660, Figure 2B). Due to this, no multivariate analysis was performed. Furthermore, no association was found regarding age or sex in both PAC and CP. In addition, there was no significant correlation between the s-ALCAM groups and the ALCAM immunohistochemical staining results (n = 39; p = 0.699).

## Discussion

The aim of the current study was to evaluate the expression and clinical significance of ALCAM in PAC tissues and to determine whether or not s-ALCAM could serve as a diagnostic and prognostic marker in the peripheral blood of PAC patients. The results showed that ALCAM was expressed in the majority of PAC lesions and that s-ALCAM serum levels were significantly elevated compared to the sera of CP patients and healthy controls (p<0.001).

The correlation between ALCAM expression in the primary PAC lesions with the clinical and pathological parameters revealed no significant findings, which confirms the results of recently published studies on smaller patient samples [29], [37]. Nonetheless, the same authors described a potential role of ALCAM in cell adhesion reduction and the induction of chemoresistance in vitro [37], and ALCAM was also described as an independent prognostic marker in PAC patients (n = 97) [29]. Contrary to the findings by Kahlert and colleagues, an overall survival analysis of our results (n = 138) showed no significant association between time of survival and the intensity and quantity of ALCAM expression (low, medium or high) (p = 0.261, Figure 2A).

The localization of ALCAM expression in pancreatic cancer cells was previously described as being mainly cytoplasmatic in PAC specimens [29]. In contrast to these results, the immunohistochemical staining protocol in the present study revealed a predominantly membranous expression of the adhesion molecule ALCAM (Figure 1) [27], [30], [31], [32]. Cytoplasmatic staining intensity was related to the intensity of the membranous staining and did not occur in the absence of membranous staining. Similar observations were made by Kristiansen and colleagues, who also found predominantly membranous staining with a variable degree of cytoplasmic staining [39]. In addition, we did not observe a membranous-cytoplasmatic shift between normal ductal cells or low grade and high grade tumors as Kahlert and colleagues did [29].

Why are there discrepancies in the morphologic and statistical results of studies investigating ALCAM in PAC? Of course, multiple factors influence the staining intensity and specificity of immunohistochemistry, and antibody concentration is only one of them. Used dilutions range from 1∶100 to 1∶450 and different pre-treatments are described in the studies, reflecting the general problem of comparability in immunohistochemical studies. Unfortunately, generally accepted guidelines for optimal immunohistochemistry protocol development are lacking [40], [41]. Recently, in a study investigating the clinical significance of p53 alterations in prostate cancer, an immunohistochemistry protocol that was deliberately designed to be “oversensitive” resulted in a much higher rate of positive immunohistochemical findings (2.5% positivity with the standard protocol compared with greater than 90% positivity with the “oversensitive” protocol) [42]. Facing this problem, our group has established a comprehensive and highly standardized protocol for an objective optimization of immunohistochemical stainings [40]. In conclusion, different protocols produce different grades of sensitivity and specificity, which leads to significant discrepancies in staining patterns and, as a result, in the statistical data. Another source for statistical differences are differences in sample sizes and divergent scoring systems. While our protocol is optimized to measure both, the quantity and tha intensity of the staining, Kahlert and colleagues have focused on intensity only [29], [40]. To preclude this as a reason for the discrepant results, we have performed an analysis using staining intensity only, which also found no statistically significant results (data not shown).

Nevertheless, not only did the majority of primary PAC lesions express ALCAM, but metastatic and recurrent lesions also showed a medium to strong expression (lymph node metastases 46%, n = 50; distant metastases 85%, n = 7; recurrent tumor lesions 67%, n = 15, data not shown). Similarly, Piscuoglio and colleagues have described a significantly elevated expression in cancer of the ampulla of Vater compared to healthy pancreatic samples and adenoma [17]. These findings would suggest a potential involvement of this factor in tumor progression of PAC [17].

This hypothesis stands in contrast to recent observations by Zhang and colleagues, who identified non small-cell lung cancer (NSCLC) stem cells, or tumor-initiating-cells by ALCAM specific FACS sorting [13]. ALCAM positive NSCLC cells showed a high proliferative potential in-vitro and in-vivo in contrast to ALCAM negative cells. Interestingly, a knock-down of ALCAM resulted not in a decrease of tumorigenicity, which is in accordance to the study by Hong and colleagues who observed similar results in an ALCAM silencing experiment of a PAC cell line via ALCAM RNAi [37]. No effect on proliferation or migration was seen. Furthermore, Zhang and colleagues presented immunohistochemical results on the impact of ALCAM tissue expression on survival in a TMA format with 143 NSCLC patients, finding no statistically significant effect. They concluded that ALCAM would be a very robust, but inert cell-surface marker for NSCLC tumor-initiating-cells. Since we have not found a significant clinical association of ALCAM expression, ALCAM might well have a similar role in PAC stem cells. If this hypothesis can be confirmed by further in-vitro and in-vivo experiments, ALCAM might become a potential target for novel antibody-based treatment strategies. The usefulness of ALCAM as a drug target structure may be further enhanced by the ligand-induced endocytosis of ALCAM [23]. In addition, a recently described internalizing single-chain antibody [23], [24], targeting ALCAM has been suggested for the potential intracellular delivery of various therapeutic agents to prostate cancer cells. However, one important objection must be raised: ALCAM is a ubiquitously expressed molecule with physiologic roles in the intestinal mucosa [43]. Severe effects on normal tissues might thus result and will have to be taken into account when an application of systemic specific cancer therapies is considered [43].

Because of the elevated expression of ALCAM in the majority of the cancerous lesions, we also evaluated the levels of s-ALCAM (from the ectodomain shedding of ALCAM) in blood sera. The s-ALCAM values were significantly elevated in PAC patients compared to patients with CP and the healthy blood donors (p<0.001 and p<0.001, respectively; Figure 3A). The AUC showed an acceptable discriminatory power of s-ALCAM (AUC = 0.690; Figure 3B). The sensitivity (58.6%) and specificity (73.9%) of s-ALCAM was clearly inferior to the tumor marker most frequently used for PAC, CA19-9, (sensitivity of 58% to 87%, specificity of 93%) [44]. However, potential methodical weaknesses of the study must be considered in the appraisal of the results: Although the handling of the serum samples is standardized at our institution, even small differences in the processing or handling can have enormous effects [45]. Moreover, the study was retrospective and conducted over a relatively long period. Hence, our results do not exclude a potential usefulness of the s-ALCAM serum quantification test, we believe the test should be further evaluated in prospective trials with larger patient groups.

In order to determine the association of elevated s-ALCAM levels with patient survival and tumor stage, different statistical analyses were performed. Neither an analysis using s-ALCAM levels as a continuous variable nor an categorical analysis with different cut-off values showed significant associations with clinical or histopathological parameters (Table 1 exemplarily shows the results of the 75^th^ percentile as cut-off level). As already mentioned, ALCAM might be an inert factor, which could explain the lack of an association [13].

Recently, a potential role of ALCAM in gemcitabine-induced chemoresistance in PAC was described, since ALCAM-silenced PAC cells showed an induced chemoresistance [37]. Investigating the clinical impact of the in-vitro data, we performed a sub-analysis of patients receiving adjuvant chemotherapy regarding survival (log-rank test). Patients with high or intermediate ALCAM expression did not show a reduced or prolonged survival compared to ALCAM negative patients (n = 60, p = 0.176). Furthermore, no survival benefit was found in patients with reduced s-ALCAM serum levels (75^th^ percentile, n = 28, p = 0.672). Due to the retrospective character of the study, few patients receiving adjuvant chemotherapy were identified, which significantly limits the power of the analysis. Moreover, different regimes were administered (Gemcitabine, Fluorouracil and others). Further analysis should be performed with larger and less heterogeneous patient cohorts, which might help to identify patients who would benefit most from an adjuvant treatment and also help tailor the best individual treatment for each patient.

The serum levels of s-ALCAM have been investigated in pancreatic cancer before, but this study was the first to analyze both ALCAM expression and s-ALCAM levels in the same patients [36], [37], [38]. Surprisingly, no significant correlation or association was found between the elevated tissue expression and serum level in the patients (n = 48, p = 0.699). Recently, s-ALCAM levels were investigated in breast and esophageal cancer patients but no correlation with tissue expression was found [30], [35]. This might have several reasons: For example, the mere expression of the ALCAM protein does not have to result in an increased shedding of s-ALCAM by proteases such as ADAM17, which was recently shown [29], [30]. Furthermore, flushing of the shedded molecule into the blood stream might be a consequence of the disruption of anatomical barriers surrounding host tissues and endothelial cells. Taken together, the mechanisms regulating the shedding of ALCAM and its dissemination into the surrounding tissue and its entry into the blood system are barely understood and further investigations are needed.

ALCAM is highly expressed in PAC specimens. The elevated ALCAM expression in primary and metastatic sites of PAC might perhaps make ALCAM a possible target for novel antibody-based treatment strategies. The immunohistochemical results of our study revealed no significant association between ALCAM expression and clinical or pathological data in PAC patients. This result is in clear contrast to further studies investigating the expression of ALCAM in pancreatic cancer and several other solid tumors of the gastrointestinal tract. Further efforts must be undertaken to investigate the physiological and oncological role of ALCAM and to validate the clinical usage of s-ALCAM as a potential clinical marker for PAC.

## Methods

### Patients and Clinical Data

For this study, tissue specimens (n = 264) and blood sera (n = 116) of patients with PAC and the sera of 115 patients with CP treated at the Department of General, Visceral and Thoracic Surgery, University Medical Center Hamburg-Eppendorf between 1993 and 2006 were analyzed. Blood samples were taken directly before surgery. None of the patients received neoadjuvant treatment. All data including sex, histology, tumor size, lymph node metastasis, disease stage (UICC 6^th^ edition). Follow-up data were obtained from a combination of clinical and pathological record reviews, from outpatient clinic medical records and communication with patients and their attending physicians, and from the cancer registry. Overall survival was calculated from the date of operation to the date of death or last follow-up. Patients who did not survive the first 30 days after surgery were excluded from the survival analysis.

The study was approved by the Ethics Committee of the Chamber of Physicians in Hamburg, Germany. Written consent for using the samples for research purposes was obtained from all patients prior to surgery or blood drawing.

### Tissue Microarray (TMA)

The pre-existing pancreatic cancer tissue microarray consists of a total of 600 tissue samples [31], [46]. These include 244 samples of primary pancreatic adenocarcinoma, 116 lymph node metastases, 12 distant metastases and 23 local recurrences from 264 patients with PAC. In addition, pancreatic tumors other than adenocarcinoma (endocrine, intraductal papillary mucinous neoplasms, benign and malignant cystic tumors, and acinar cell tumors), a standard control area containing 40 tumors from other organs, healthy pancreatic tissues, and 18 healthy tissues from other sites are included. Construction of the TMA was described previously [47]. Briefly, haematoxylin-eosin stained sections were made from selected primary tumor blocks (donor blocks) to define representative tumor regions. Tissue cylinders (0.6 mm in diameter) were then punched from that region of the donor block using a home-made semi-automated tissue arrayer. Sections of 3 µm in size were cut using the Paraffin Sectioning Aid System (Instrumentics, Hackensack, NJ, USA).

### Immunohistochemical Staining for ALCAM and Evaluation of ALCAM Expression

The ALCAM staining protocol was optimized in an extensive and standardized multi-step procedure on various benign and malignant tissues; the protocol was modified until selective staining with the lowest background signals was established [40]. Freshly cut TMA sections were analyzed on one day in a single experiment. The expression of ALCAM was detected using a mouse monoclonal antibody (clone MOG/07, 1∶450; Novocastra, Newcastle upon Tyne, UK) after the sections had been covered with a citrate buffer, pH 7.8, and boiled in an autoclave. The EnVision system (Dako, Glostrup, Denmark) was used to visualize the immunostaining.

Only membranous staining was evaluated because cytoplasmatic staining – if present – was always linked to stronger membranous staining. The staining intensity (0, 1+, 2+, 3+) and the fraction of positive tumor cells were scored for each tissue spot as recently published [40]. Spots without staining and with a staining intensity of 1+ in <70% and 2+ in <30% of the tumor cells were scored as ALCAM low, medium scores were given for a staining intensity of 1+ in ≥70%, 2+ in ≥30% or 3+ in <30% of the tumor cells, and high scores were given for a staining intensity of 2+ in ≥70% or 3+ in ≥30% of the tumor cells. Immunohistochemical analysis of the sections was performed without knowledge of the patients’ identity or clinical status.

### Sandwich ELISA for the Detection of s-ALCAM

For s-ALCAM quantification in peripheral blood, serum samples of 116 Caucasian patients with PAC and 115 Caucasian patients (37 female and 78 male, median age 50.1 years (31.4–79.2 years)) with CP, who were indicated for surgical treatment, were analyzed with an s-ALCAM sandwich enzyme linked immunoassay (ELISA). All blood samples were obtained directly before surgery. As healthy controls, 128 Caucasian blood-bank donors (62 female and 66 male, median age 48.7 years 19.2–65.3 years) were included in the study. All sera were processed latest after 4 hours [45].

For the detection of s-ALCAM, flexible 96-well microtiter plates (Costar, Corning, NY, USA) were coated with 50 µl per well of 2 µg/ml of monoclonal mouse capturing antibody (MAB6561; R&D Systems, Minneapolis, MN, USA) overnight at 4°C. The wells were blocked with 3% w/v bovine serum albumin (BSA; Fraktion V, 98% purity; Sigma Aldrich, Taufkirchen, Germany) in PBS/T (PBS pH 7.3 containing 0.05% v/v Tween) for 45 min at room temperature, and then incubated for 1 h at room temperature with human serum samples diluted 1∶50 in PBS. After five washes with PBS/T, bound protein was detected by a biotin-conjugated polyclonal antibody (BAF656; R&D Systems), followed by streptavidin-conjugated peroxidase using 3,3',5,5?-tetramethylbenzidine (TMB) as the substrate. The color reaction was stopped by the addition of 10 mM H_2_SO_4_, and analyzed at 450 nm using an ELISA reader (Dynatech MR 5000; Pegasus Scientific, Rockville, MD, USA). Human Alcam–Fc protein (R&D Systems) served as an internal standard for the assay.

In order to ensure that the immunoassay was suitable for measuring clinical serum samples, reproducibility and linearity were examined. The assay showed excellent linearity with serial dilutions and showed <10% coefficient of variation (CV) for the intra- and inter-assay variability studies.

### Statistical Analysis

The statistical analysis was performed using SPSS Statistics for Windows (Version 17, SPSS Inc., Chicago Ill, USA). Interdependence between immunostaining and ELISA results as well as the clinical data was calculated using Chi-square and Fisher’s exact tests and displayed in cross tables. The cut-off level for s-ALCAM quantification was determined using the Youden-index. Group differences were calculated by the t-test, ANOVA; Mann-Whitney or Kruskal-Wallis test. Receiver operating characteristic curves were used to describe the performance of s-ALCAM. Survival curves were plotted using the Kaplan-Meier method and analyzed using the log-rank test. All tests were two-sided and p-values less than 0.05 were considered statistically significant.
